# Special Issue: Design and Control of a Bio-Inspired Robot

**DOI:** 10.3390/biomimetics9010043

**Published:** 2024-01-10

**Authors:** Mingguo Zhao, Biao Hu

**Affiliations:** 1 Department of Automation, Tsinghua University, Beijing 100084, China; mgzhao@mail.tsinghua.edu.cn; 2 College of Engineering, China Agricultural University, Beijing 100083, China

## 1. Introduction

Bionics, the interdisciplinary field that draws inspiration from nature to design and develop innovative technologies, has paved the way for the creation of “bio-inspired robots”. These remarkable machines simulate and replicate biological mechanisms to achieve specific functions, presenting a fascinating fusion of biology and engineering. This special issue delves into the various facets of bionics in robotics such as bionics in robot design, perception, control and decision-making, and incorporating neuroscience and brain science. It also explores the recent advancements that promise to reshape the landscape of robotic intelligence and application scenarios. This paper collection covers a wide range of topics in robotics, including stiffness adjustment for continuum robots, biomimetic motor control, stroke rehabilitation, reinforcement learning for quadruped robots, improved spiking neural networks, energy-efficient image segmentation, kinematics analysis, synthetic nervous systems for robotic control, online running-gait generation, and bio-inspired perception and navigation for service robots.

## 2. Discussion of the Papers

Aiming to examine robotic perception and navigation techniques in indoor environments and addressing potential research challenges, the review paper on bio-inspired perception and navigation for service robots by Jianguo Wang and coworkers (2023) [1] reviews bio-inspired perception and navigation for service robots in indoor environments. It classifies navigation approaches by perception type and highlights the trend towards multi-modal navigation. Challenges include precise localization and navigating dynamic, complex environments with moving objects and people.

Legged robots face multiple challenges including stability, energy efficiency, and complex control due to the need to coordinate multiple limbs and joints. These robots must adapt to different terrains, navigate obstacles, and maintain robustness and durability. Achieving high levels of autonomy and ensuring safe interaction with humans are ongoing challenges in the field of legged robotics. Three contributions in this Special Issue deal with these problems. The paper by Peng Sun et al. (2023) [2] conducts a kinematics analysis of a hybrid mechanical leg for bipedal robots and develops a gait planning strategy based on the inverted pendulum model. Dynamic simulations confirm the robot’s stable walking on flat ground, providing insights into gait planning for such robots. This research offers valuable insights into gait planning for hybrid mechanical legged bipedal robots, serving as a foundational reference for future research in this domain. The paper by Xiang Meng et al. (2023) [3] presents an online running-gait generator for bipedal robots, focusing on smooth state switching and accurate speed tracking. It utilizes a variable-height inverted-pendulum model, segmented zero moment point (ZMP) trajectory optimization, and an iterative algorithm to achieve stable running and precise speed tracking. Experimental results using the BHR7P bipedal robot validate the effectiveness of these proposed methods. The paper by Yanbiao Li et al. (2023) [4] presents a hierarchical reinforcement learning framework based on the Deep Deterministic Policy Gradient (DDPG) algorithm. This framework involves a high-level planner that generates ideal motion parameters, a low-level controller using model predictive control (MPC), and a trajectory generator. The high-level planner trains agents to provide optimal motion parameters for the low-level controller, which uses MPC and PD controllers to generate foot-end forces and calculate joint motor torque through inverse kinematics. Simulation results demonstrate the superior motion performance achieved by this hierarchical framework compared to using the DDPG method alone.

Motion control of continuum robots and robotic arms presents several challenges. Firstly, their inherent flexibility and compliance make high-precision motion control difficult, requiring innovative stiffness adjustment mechanisms. Secondly, achieving coordinated and precise multi-degree-of-freedom motion in robotic arms demands advanced control algorithms and sensor integration. Lastly, real-time control and adaptation to changing environments are essential, posing challenges in perception, planning, and control for both types of robots. Three contributions in this Special Issue deal with these challenges. The paper by Mingyuan Wang et al. (2023) [5] introduces a Stiffness Adjustment Mechanism (SAM) integrated into a growth-controllable continuum robot (GCCR) to enhance its motion accuracy in variable-scale motion. SAM uses antagonistic cable force transmission to self-adjust stiffness during length changes. The static model accounts for gravity and end loads, providing reliable predictions of robot shape deformations. Experimental validation confirms a maximum error ratio within 5.65% and a stiffness enhancement of nearly 79.6%. The paper by Qingkai Li et al. (2023) [6] presents a central nervous system-based Biomimetic Motor Control (CBMC) approach with four modules for controlling a 7-DoF robotic arm. It includes spiking neural networks, artificial neural networks, cerebral sensory, and spinal cord modules. CBMC is validated for trajectory tracking and exhibits effectiveness in various scenarios, including different payloads. The paper by Jong-Chen Chen et al. (2023) [7] utilizes an Artificial Neuromolecular System (ANM) to control a mechanical arm for stroke patients’ rehabilitation. ANM captures spatiotemporal information and enables continuous learning, dimensionality reduction, transfer learning, and fault tolerance. The results demonstrate improved arm movement trajectories, reduced muscle activation, and faster action switching for stroke patients.

Improving the robot perception by bio-inspired neural network is another important research topic. Compared to other neural network such as convolutional neural networks, spiking neural networks (SNNs) are biologically inspired, closely mirroring the functioning of real neurons, which can lead to better insights into brain-inspired computation. In addition, SNNs are inherently more energy-efficient due to their event-driven, sparse firing mechanism, making them well-suited for low-power and neuromorphic hardware applications. SNNs excel in processing temporal information with precise spike timing, enabling them to perform exceptionally well in tasks involving dynamic or time-sensitive data. Three contributions in this Special Issue focus on SNN. The paper by Xiongfei Fan et al. (2023) [8] introduces a novel approach called IDSNN (initialization and distillation for SNNs) to address the low accuracy and high inference latency faced by SNNs. IDSNN leverages parameter initialization and knowledge distillation from ANNs, resulting in competitive top-1 accuracy for CIFAR10 (94.22%) and CIFAR100 (75.41%) with minimal latency. Importantly, IDSNN demonstrates a remarkable 14× faster convergence speed compared to direct SNN training, making it a valuable tool for practical applications with limited training resources. The paper by Hong Zhang et al. (2023) [9] presents the development of a Spiking Context Guided Network (Spiking CGNet) capable of efficient segmentation for both frame and event-based images. The paper outlines a spiking context guided block for local feature extraction and context information using spike computations. Two variants of the network, SCGNet-S and SCGNet-L, are established for different image types. The results demonstrate the superior energy efficiency of SCGNet-S compared to ANN CGNet on the Cityscapes dataset and the outperformance of Spiking CGNet on the DDD17 dataset compared to other spiking segmenters. The paper by William R. P. Nourse et al. (2023) [10] introduces SNS-Toolbox, an open-source Python package for simulating networks of dynamic neurons and conductance-based synapses, known as synthetic nervous systems. It supports hundreds to thousands of spiking and non-spiking neurons, enabling real-time simulations for robotic control applications.

## 3. Conclusions

Due to the success of this first special issue of “Design and Control of a Bio-Inspired Robot”, the guest editor team intends to call for a second Special Issue. This new Special Issue will still accept manuscripts on the following: (1) biomechanical-inspired locomotion; (2) neuromorphic sensing; (3) muscle-like actuators; (4) bionic visual perception; (5) biofeedback control; (6) swarm intelligence; (7) bio-inspired navigation; (8) biological sensory fusion; (9) morphological adaptation and (10) evolutionary algorithms.
